# Downstroke and upstroke conflict during banked turns in butterflies

**DOI:** 10.1098/rsif.2021.0779

**Published:** 2021-12-01

**Authors:** P. Henningsson, L. C. Johansson

**Affiliations:** Department of Biology, Lund University, Ecology Building, Sölvegatan 37, Lund 223 62, Sweden

**Keywords:** butterflies, aerodynamics, manoeuvring, flight

## Abstract

For all flyers, aeroplanes or animals, making banked turns involve a rolling motion which, due to higher induced drag on the outer than the inner wing, results in a yawing torque opposite to the turn. This adverse yaw torque can be counteracted using a tail, but how animals that lack tail, e.g. all insects, handle this problem is not fully understood. Here, we quantify the performance of turning take-off flights in butterflies and find that they use force vectoring during banked turns without fully compensating for adverse yaw. This lowers their turning performance, increasing turn radius, since thrust becomes misaligned with the flight path. The separation of function between downstroke (lift production) and upstroke (thrust production) in our butterflies, in combination with a more pronounced adverse yaw during the upstroke increases the misalignment of the thrust. This may be a cost the butterflies pay for the efficient thrust-generating upstroke clap, but also other insects fail to rectify adverse yaw during escape manoeuvres, suggesting a general feature in functionally two-winged insect flight. When lacking tail and left with costly approaches to counteract adverse yaw, costs of flying with adverse yaw may be outweighed by the benefits of maintaining thrust and flight speed.

## 1. Introduction

Manoeuvring flight involves generating aerodynamic torques and forces that allow for a change in heading or speed. Compared to aeroplanes manoeuvring, these tasks are complicated by the flapping of wings in many animals. In addition to enhancing passive counter roll and yaw torques resisting aerodynamic manoeuvres (e.g. [1]), flapping also allows for mechanisms not seen in aeroplanes to accomplish manoeuvres, e.g. differential thrust between wings [2], changing stroke plane to direct the force [3] and, in birds and bats, altering wing area and span by folding a wing [2] The knowledge of how various animal groups handle manoeuvres and what performance trade-offs may exist is limited.

To reduce the complexity of flapping flight manoeuvres, we focus on insects where wingspan and area are fixed throughout the wingbeat. Studies of aerodynamics of manoeuvring flight in insects have mainly focused on flies [4–7] or other fast flapping insects with moderate to high aspect ratio wings (e.g. moths, e.g. [8,9]), but see [10] for an exception. Here, we will instead focus on a group of insects with low aspect ratio wings flapped at relatively low frequency, namely butterflies. Butterflies have been shown to have distinct separation in function between downstroke and upstroke, at least during take-off, where the downstroke is used for weight support and the upstroke [11,12], with the distinct wing clap, for thrust [11]. How this separation affects the ability to perform turns is unknown, but we hypothesize that it may limit the performance of certain types of manoeuvres, for example banked turns. Banked turns are initiated, in both animals and aeroplanes, by a rolling motion, created by the outer wing producing relatively more lift than the inner wing [13], which results in an additional-induced drag on the outer wing creating a yawing torque and rotation opposite to the turn, called adverse yaw [13]. This occurs for all flyers, unless the yaw torque is countered [13], and results in a slideslip. Aeroplanes, birds and bats use their tail to counteract adverse yaw [13,14]. Butterflies, and other insects, do not have tails and hence adverse yaw needs to be controlled differently. One way to counter adverse yaw is to increase thrust on the outer wing-pair (hereafter referred to simply as ‘wing’) during downstroke or, alternatively, generate more drag on the inner wing during either downstroke or upstroke. However, in strong turns, flies have been shown to ignore, or not fully compensate for, the adverse yaw while performing the manoeuvre and only correct it at the end of the turn [15]. How, or if, butterflies rectify adverse yaw during banked turns is, as far as we know, unknown.

Here, we use previously published data [11] to conduct a new analysis for studying the aerodynamics and kinematics of banked take-off turns in a butterfly species, silver-washed fritillary (*Argynnis paphia)*, to determine how they accomplish the manoeuvres and deal with adverse yaw.

## 2. Material and methods

We used kinematics (from high-speed videos) and aerodynamics (from tomoPIV) raw data of six silver-washed fritillaries in take-off flights in a wind tunnel [11] set at approximately 2 m s^−1^. See electronic supplementary material information for details on the experimental procedure, data processing and analysis.

In addition to data presented in [11], we determined wingbeat average vertical and side forces from the wake and required centripetal force from kinematic data. We also calculated aerodynamic lift (perpendicular to the wake bank angle, electronic supplementary material, figure S1B) for the inner and outer wing separately. We measured bank and yaw angle relative to the flight path (through still air) and peak angle of attack of the wings during downstroke from the kinematic data. For statistics, we used mixed general linear models in JMP Pro 15.0.0 (SAS Institute Inc., Cary, USA) to address the repeated measures set-up. All results include 95% confidence intervals (CIs). For details, see electronic supplementary material.

## 3. Result and discussion

**Figure 1.**
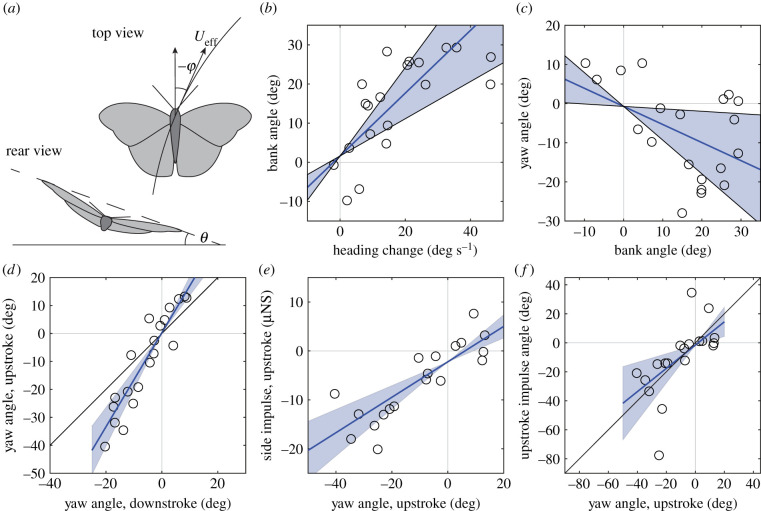
Kinematic and aerodynamic results from banked turns in silver-washed fritillaries. (*a*) Bank angle (*θ*), defined as the angle between a line connecting the wing-tips and the horizon, and yaw angle (*φ*) as the angle between a line perpendicular to the wing-tip line and the tangent of the horizontally projected path of the turn. *U*_eff_ is the flight speed. (*b*) Bank angle is positively correlated (*p* < 0.0001) with speed of change of heading ($\dot{\gamma }$) (Blue line: *θ* = 0.80 (CI ± 0.33) * $\dot{\gamma }+1.62(\mathrm{CI}\pm 8.36)$, *r*^2^ = 0.67). For this and the following panels, shaded areas indicate CI of the slope of the fitted lines. (*c*) Yaw angle was negatively correlated with bank angle (*p* = 0.024) i.e. showing an uncorrected adverse yaw, but with rather large variation (blue line: *φ* = –0.46 (CI ± 0.4) * *θ* –0.72 (CI ± 7.59), *r*^2^ = 0.17). (*d*) We found a positive relation between the yaw angle during the downstroke (*φ*_d_) and the upstroke (*φ*_u_) (*p* < 0.0001) with a stronger adverse yaw during upstroke than during downstroke (blue line: *φ*_u_ = 1.68 (CI ± 0.35) * *φ*_d_ + 0.25 (CI ± 3.62), *r*^2^ = 0.75), differing significantly from the predicted 1 : 1 relation (black line). (*e*) Yaw angle during upstroke correlated well with average side impulse generated during upstroke (*I*_u_) (*p* < 0.001) (blue line: *I*_u_ = 3.62 × 10^−7^ (CI ± 1.19 × 10^−7^) * *φ*_u_ – 2.22 × 10^−6^ (CI ± 2.27 × 10^−6^), *r*^2^ = 0.58), where upstroke impulse acted in the opposite direction to the required centripetal force. (*f*) Yaw angle during upstroke determines the direction of impulse generated during upstroke (*φ*_Iu_) (*p* = 0.002) (blue line: *φ*_Iu_ = 0.80 (CI ± 0.51) * *φ*_u_ −1.60 (CI ± 12.3), *r*^2^ = 0.38), which does not differ from the expected 1 : 1 relation (black line). Regressions are from a mixed linear model taking into account repeated measures within individuals.

During take-off turns, our butterflies banked to accomplish the manoeuvre (figure 1*b*), where measured side force matched required centripetal force (electronic supplementary material, figure S2) demonstrating that they use force vectoring to accomplish the turn. We also found that the butterflies did not correct an adverse yaw present during the turn (figure 1*c*). The adverse yaw resulted in the upstroke thrust being misaligned with the flight direction (figure 1*e,f*). The upstroke thus counteracted the downstroke, producing a side force in the opposite direction to that of the downstroke (e.g. during a turn to the right thrust will push to the left), reducing turning performance of the take-off manoeuvre. The cost, a relative reduction in turning performance, may be the price paid for thrust production during the upstroke. However, the effect on performance may be small given that a flapping robot adjusting yaw did not improve time for completing turns [15], although a direct inference to butterflies may be problematic due to differences in the aerodynamic function of their wings.

**Figure 2.**
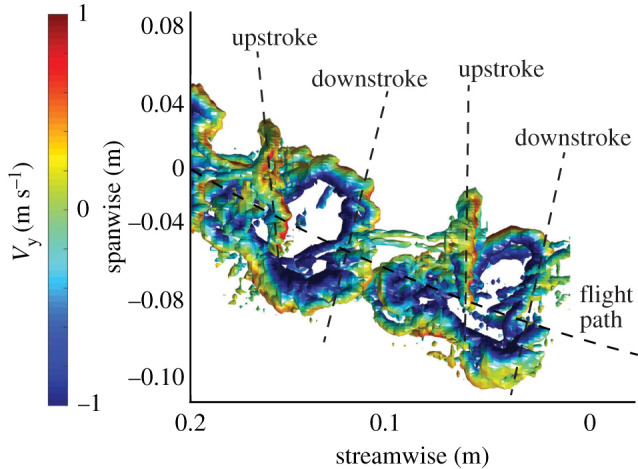
Vortex wake of a butterfly during a banked turn. The wake, seen from above with flight direction to the left, illustrates variation in yaw angle of the wake between downstroke and upstroke, as indicated by the dashed lines. The upstroke wake indicates force production in the horizontal plane, perpendicular to the dashed line, resulting in thrust and sideways force opposite to the centripetal force required to conduct the turn. Vortices are shown as iso-surfaces of q-criterion coloured by downwash velocity (blue downwards flow and red upwards).

We found that adverse yaw was more pronounced during upstroke than downstroke (figure 1*d* and figure 2), suggesting yaw changes dynamically through the wing stroke, something also suggested in cicadas [10]. Conventionally viewed, adverse yaw acts only during the initial rolling motion of banked turns and when the desired bank angle is achieved, lift is symmetrical and adverse yaw torque ceases. That we see adverse yaw varying between up and downstroke in our butterflies suggests that some additional mechanism is at work. The velocity difference between the two wings, where during a tight turn the outer wing will move faster through the air than the inner wing and generate a higher profile drag, will for example result in an adverse yaw torque.

Counteracting adverse yaw without a tail can be done through producing relatively more thrust (or less drag, which is not likely if lift is maintained) on the outer wing. Since downstroke in our butterflies does not contribute to thrust, a corrective force must come from the upstroke and clap, which may be facilitated by body pitch changes between downstroke and upstroke [16], or as more drag on the inner wing during the downstroke. In butterflies, upstroke thrust is generated by two mechanisms, where the initial phase uses a drag-based mechanism and the late stages a wing clap. The latter depends to a large extent on the speed at which the wings reduce space between them [11] which should have little potential to influence the yaw torque, since the two wings together create the thrust. The drag-based mechanism, on the other hand, depends on the wing speed relative to air, which is a combination of wing flapping speed and flight speed. In a banked turn, the inner wing will experience relatively lower flight speed, so if the wings are flapped backwards at equal speed, the inner wing will generate relatively more thrust, resulting in a torque enhancing adverse yaw. So, not only does thrust produced during upstroke in an adverse yaw situation act to increase the radius of the turn, it may also act to increase adverse yaw itself. This could explain why we find stronger adverse yaw during upstroke than downstroke and suggests a higher cost for our butterflies using the wing clap to boost thrust than for insects not using the wing clap.

There may, however, be other factors affecting the results. The higher speed experienced by the outer wing than the inner wing will, all else being equal, results in more lift generated on the outer wing during downstroke and hence a roll torque enhancing bank angle. To stabilize the bank angle, butterflies may increase lift on the inner wing by either flapping it at increased amplitude and/or increased angle of attack. We did not find a difference in amplitude between the wings (*A*_o_/*A*_I_ = 1.02, *p* = 0.35, CI 0.98–1.06), but found higher peak angles-of-attack on the inner wing during downstroke (*α*_o_ − *α*_i_ = −5.4 [CI −7.5 to −3.2] degrees, *p <* 0.0001) suggesting our butterflies try to maintain bank angle during the turn. This is further supported by the fact that we found no difference in aerodynamic lift between the wings during the wingbeat (*L*_aero,o_/*L*_aero,i_ = 1.11, *p* = 0.088, CI 0.98–1.25). One effect of increasing angle of attack on the inner wing is that induced and profile drag will increase, causing a yaw torque that will counter adverse yaw. Using drag on the inner wing to generate yaw is not unique to our butterflies but also found in other animals (i.e. drone flies [5] and bats [2]). If the increase in angle of attack on the inner wing is restricted by lift production (excessive lift would roll the animal out of the banked turn), the added drag may be below the necessary amount to fully rectify the adverse yaw. Taken together, the downstroke counteracts adverse yaw without fully rectifying it, while the upstroke tends to increase adverse yaw—and hence we find stronger adverse yaw during upstroke compared to downstroke.

The fact that insects with as diverse flight styles and wing morphology as our butterflies and fruit flies [4] do not rectify adverse yaw during escape/take-off manoeuvres suggests the potential of a general feature of insect flight, or at least functionally two-winged species. When lacking a tail and left with costly approaches to rectify adverse yaw, costs of flying with adverse yaw may be outweighed by the benefits of maintaining thrust and flight speed.

## Data accessibility

The vector data that support the finding of this study are available from the Dryad Digital Repository: https://doi.org/10.5061/dryad.4j0zpc87v [17]. The Matlab codes used to analyse the data will be available from the authors upon request. The data are provided in the electronic supplementary material [18].
